# Pogostone inhibits the activity of CYP3A4, 2C9, and 2E1 *in vitro*

**DOI:** 10.1080/13880209.2021.1917630

**Published:** 2021-04-29

**Authors:** Guiying Zhang, Yanping Zhang, Xianjie Ma, Xin Yang, Yuyan Cai, Wenli Yin

**Affiliations:** a Department of Pharmacy, People’s Hospital of Rizhao, Rizhao, China; b Department of Pediatrics, People’s Hospital of Rizhao, Rizhao, China

**Keywords:** CYP450 enzymes, drug-drug interaction, CYP2C9, CYP2E1

## Abstract

**Context:**

Pogostone possesses various pharmacological activities, which makes it widely used in the clinic. Its effect on the activity of cytochrome P450 enzymes (CYP450s) could guide its clinical combination.

**Objective:**

To investigate the effect of pogostone on the activity of human CYP450s.

**Materials and methods:**

The effect of pogostone on the activity of CYP450s was evaluated in human liver microsomes (HLMs) compared with blank HLMs (negative control) and specific inhibitors (positive control). The corresponding parameters were obtained with 0–100 μM pogostone and various concentrations of substrates.

**Results:**

Pogostone was found to inhibit the activity of CYP3A4, 2C9, and 2E1 with the IC_50_ values of 11.41, 12.11, and 14.90 μM, respectively. The inhibition of CYP3A4 by pogostone was revealed to be performed in a non-competitive and time-dependent manner with the *K_i_* value of 5.69 μM and the KI/K_inact_ value of 5.86/0.056/(μM/min). For the inhibition of CYP2C9 and 2E1, pogostone acted as a competitive inhibitor with the *K_i_* value of 6.46 and 7.67 μM and was not affected by the incubation time.

**Discussion and conclusions:**

The inhibitory effect of pogostone on the activity of CYP3A4, 2C9, and 2E1 has been disclosed in this study, implying the potential risk during the co-administration of pogostone and drugs metabolized by these CYP450s. The study design provides a reference for further *in vivo* investigations to validate the potential interaction.

## Introduction

Pogostone is a major component of *Pogostemon cablin* (Blanco*)* Benth. (Lamiaceae), which is known as ‘Guang-Huo-Xiang’ in Chinese. Pogostone was reported to possess various pharmacological activities, including antibacterial (Osawa et al. 1990), anti-inflammatory (Chen et al. 2015), antifungal (Peng et al. 2014), and immunosuppressive activities (Su et al. 2015). Previously, pogostone has been demonstrated to protect against acute lung injury and lung cell injury (Sun et al. 2016; Yang et al. 2018). Due to the increasing significance of pogostone as a bacteriostat, pogostone has been commonly used in the clinic. Combing two or more different types of herbs or drugs is a commonly used prescription to improve therapeutic efficiency and achieve better treatment.

Drug-drug interaction between drugs or herbs is one of the primary factors that induces adverse effects, even failed treatment in the clinic. Cytochrome P450 enzymes (CYP450s) is a superfamily that contains heme-thiolate proteins and functions as monooxygenases, which are involved in the metabolism of various drugs (Wrighton and Stevens 1992). Numerous studies have suggested that CYP450s are key factors that mediate drug-drug interaction as the inhibition or induction of CYP450 activity could affect the metabolism of drugs. For example, the co-administration of glycyrrhizin and asiatic acid could reduce the plasma concentration of asiatic acid and shorten its half-life due to the induced effect of glycyrrhizin on the activity of CYP3A4, which is responsible for the metabolism of asiatic acid (Guo et al. 2018). Berberine could inhibit the pharmacokinetics of losartan, decrease the concentration of EXP3174 and the metabolite of losartan through inhibiting the activity of CYP3A4 or 2C9 (Li et al. 2016).

A variety of drugs have been reported to inhibit or induce the activity of CYP450s, such as dihydromyricetin (Liu et al. 2017), cepharanthine (Zhang et al. 2020), and paroxetine (Kamel and Lamsabhi 2020). Here, the effects of pogostone on the activity of CYP1A2, 2A6, 2A4, 2C8, 2C9, 2C19, 2D6, and 2E1 were investigated *in vitro* in pooled human liver microsomes. The activity of CYP450s was estimated by probe substrates and marker reactions to provide reference and guidance for the clinical co-administration of pogostone.

## Materials and methods

### Chemicals

Pogostone was obtained from the National Institutes for Food and Drug Control (Beijing, China). The substrates, including phenacetin, testosterone, coumarin, chlorzoxazone, dextromethorphan, diclofenac, mephenytoin, and paclitaxel, were obtained from Sigma Chemical Co. (Chicago, IL, USA). Pooled human liver microsomes (HLMs) were purchased from BD Biosciences (Woburn, MA, USA). The purity of chemicals was more than 98%, and other reagents were of analytical reagent grade.

### Assay with HLMs

The effect of pogostone on the activity of CYP450s was investigated with probe reactions (summarized in Table 1) in HLMs in accordance with previous studies (Zhang et al. 2007, 2020). All incubations were conducted in triplicate with a volume of 200 μL, containing 100 mM potassium phosphate buffer (pH 7.4), NADPH-generating system (1 mM NADP^+^, 10 mM glucose-6-phosphate, 1 U/mL of glucose-6-phosphate dehydrogenase, and 4 mM MgCl_2_), corresponding probe substrates, HLM, and 100 μM pogostone or positive inhibitors. The concentrations of positive inhibitors were: 10 μM furafylline for CYP1A2, 1 μM ketoconazole for CYP3A4, 10 μM tranylcypromine for CYP2A6, 50 μM clomethiazole for CYP2E1, 10 μM quinidine for CYP2D6, 10 μM sulphaphenazole for CYP2C9, 50 μM tranylcypromine for CYP2C19, and 5 μM montelukast for CYP2C8. The microsome protein concentration and other incubation conditions were summarized in Table 1.

**Table 1.** Isoforms tested, marker reactions, incubation conditions, and K_m_ used in the inhibition study.

CYPs	Marker reactions	Substrate concentration (μM)	Protein concentration (mg/mL)	Incubation time (min)	Estimated K_m_ (μM)
1A2	phenacetin *O*-deethylation	40	0.2	30	48
2A6	coumarin 7-hydroxylation	1.0	0.1	10	1.5
3A4	testosterone 6β-hydroxylation	50	0.5	10	53
2C8	paclitaxel 6α-hydroxylation	10	0.5	30	16
2C9	diclofenac 4′-hydroxylation	10	0.3	10	13
2C19	*S*-Mephenytoin 4-hydroxylation	100	0.2	40	105
2D6	dextromethorphan *O*-demethylation	25	0.25	20	4.8
2E1	chlorzoxazone 6-hydroxylation	120	0.4	30	126

A 3 min preincubation was performed at 37 °C before the reactions were initiated by the addition of the NADPH-generating system. After the incubation, 100 μL acetonitrile or 10% trichloroacetic (for CYP2A6) was added to terminate the reactions and placed on ice. The mixture was centrifuged at 12,000 rpm for 10 min, and an aliquot (50 μL) of supernatant was transferred for corresponding metabolites analysis with HPLC.

### Enzyme inhibition and kinetic studies

The effect of pogostone on the activity of CYP450s was first investigated with 100 μM pogostone in HLMs. Then, the CYPs, of which the activity was inhibited by pogostone were incubated with 0, 2.5, 5, 10, 25, 50, and 100 μM pogostone and different probe substrates (20, 40, 60, and 100 μM testosterone for CYP3A4, 5, 10, 15, and 20 μM diclofenac for CYP2C9, or 25, 50, 150, and 250 μM chlorzoxazone for CYP2E1) to obtained the corresponding kinetic parameters, such as IC_50_ and K_i_ values.

### Time-dependent inhibition study

The effect of incubation on the inhibitory effect of pogostone was evaluated by incubating for 0, 5, 10, 15, and 30 min at 37 °C. The concentration of pogostone in the time-dependent inhibition study was 20 μM with the 1 mg/mL HLMs. After incubation, an aliquot (20 μL) was transferred to another incubation tube (a final volume of 200 μL) containing an NADPH-generating system and probe substrates whose final concentrations were approximate to *K_m_*. Then, further incubations were performed to measure the residual activity. After being incubated for 10 and 30 min, the reactions were terminated by adding a 100 μL acetonitrile internal standard mix and then placed on ice; the corresponding metabolites were determined by HPLC.

Further experiments were performed to quantify the time-dependent inhibition by the KI and Kinact values. The incubation was conducted with a higher substrate concentration, which was about 4-fold to Km and 0, 2, 5, 10, 20, and 50 μM pogostone for 0, 5, 10, 15, and 30 min. A two-step incubation scheme was performed as described above.

### Statistical analysis

The Lineweaver-Burk plots were used to obtain enzyme kinetic parameters by the least-squares linear regression of the inverse substrate concentration versus the inverse velocity. Inhibition data from the experiments that were conducted using multiple compound concentrations were represented by Dixon plots, and inhibition constant (Ki) values were calculated using non-linear regression according to the following equation:

$$V=(V_{\max}S)/(K_{m}(1+I/K_{i})+S)$$

where I is the concentration of the compound, *K_i_* is the inhibition constant, S is the concentration of the substrate and *K_m_* is the substrate concentration at half the maximum velocity (*V_max_*) of the reaction. The mechanism of the inhibition was inspected using the Lineweaver–Burk plots and the enzyme inhibition models. The data comparison was performed using the Student’s *t*-test and performed using IBM SPSS statistics 20 (SPSS Inc., Chicago, IL, USA).

## Results

### Effect of pogostone on the activity of CYP450s

**Figure 1.**
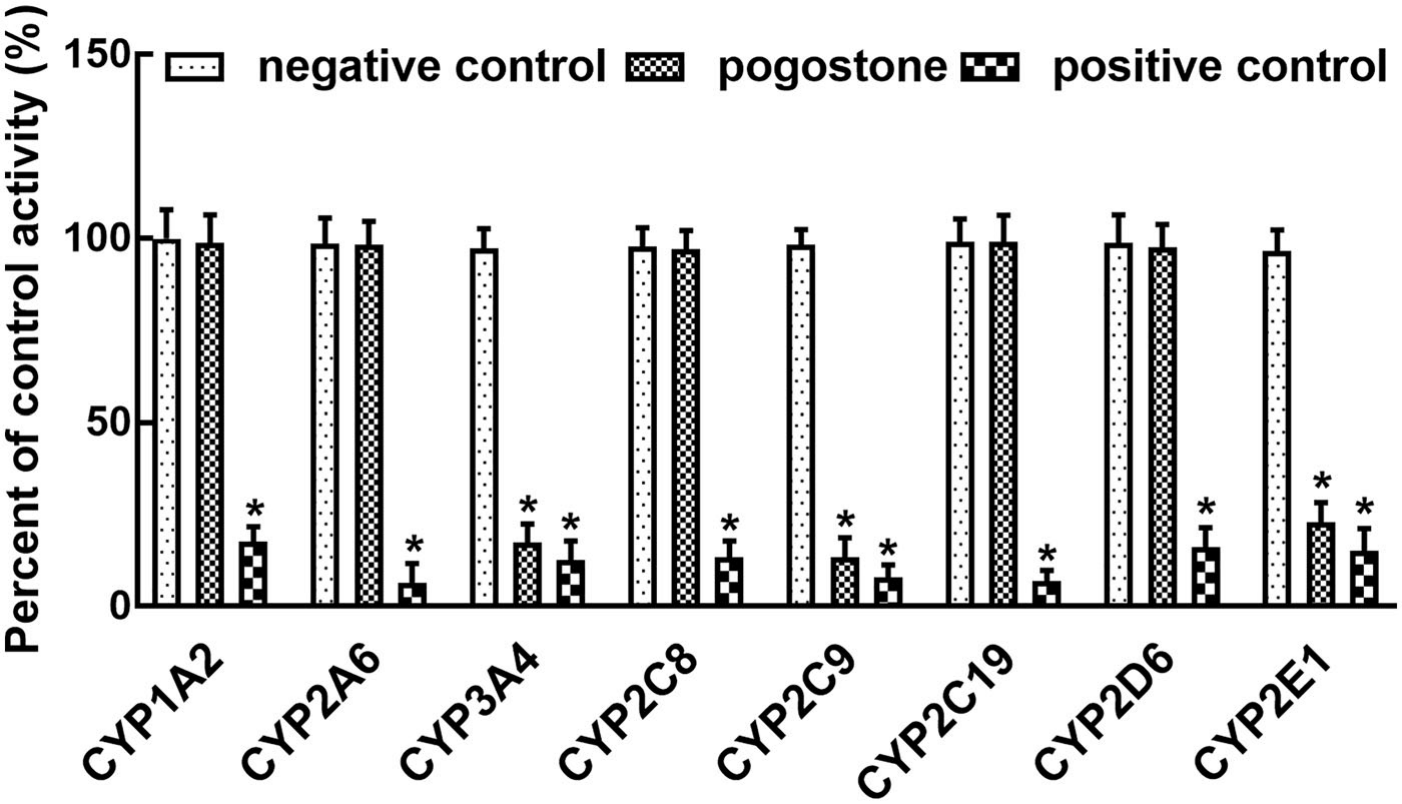
Effect of pogostone on the activity of CYP450s. The activity of CYP3A4, 2C9, and 2E1 was inhibited by pogostone. Negative control: without pogostone or positive inhibitors; Pogostone: 100 μM pogostone; Positive control: corresponding positive inhibitors. *p < 0.05.

Corresponding positive inhibitors dramatically inhibited the activity of all isoforms of CYPs (*p* < 0.05, Figure 1). In the presence of pogostone, the activity of CYP3A4, 2C9, and 2E1 was significantly inhibited compared with negative controls (*p* < 0.05, Figure 1). The inhibitory effect of pogostone was weaker than that of positive inhibitors. Through dose-dependent studies with the concentration of pogostone of 0, 2.5, 5, 10, 25, 50, and 100 μM, the IC_50_ values of CYP3A4, 2C9, and 2E1 were obtained as 11.41, 12.11, and 14.90 μM.

### The inhibition of CYP3A4 by pogostone

**Figure 2.**
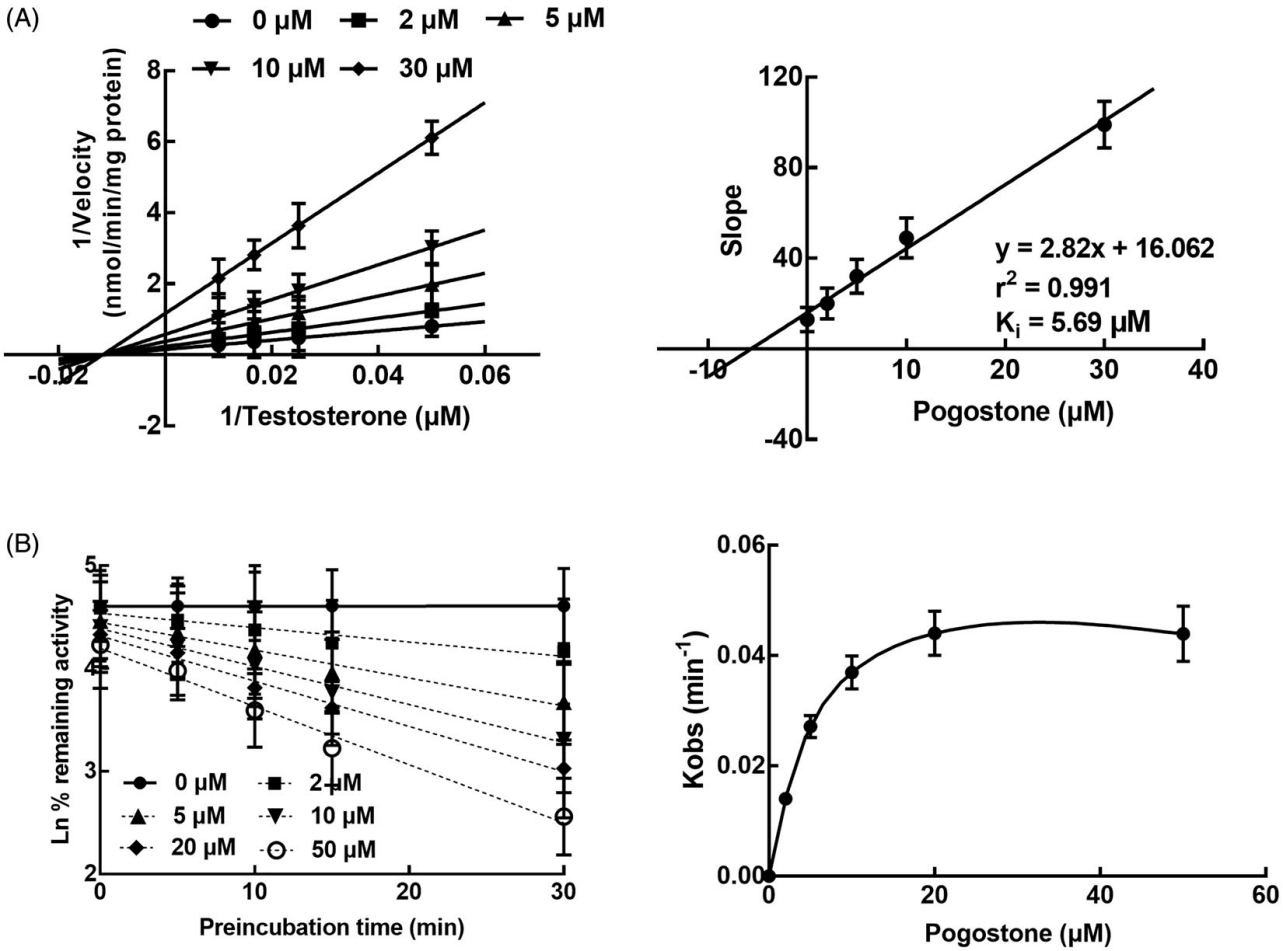
The inhibition of CYP3A4 by pogostone. A. The Lineweaver-Burk plots of CYP3A4 in the presence of 0, 2, 5, 10, and 30 μM pogostone and 20–100 μM testosterone. The inhibition of CYP3A4 was found to be non-competitive and the *K_i_* value was obtained as 5.69 μM. B. The inhibition of CYP3A4 was affected by the incubation time and the *KI* and *K_inact_* value were obtained as 5.86/μM and 0.056/min, respectively.

The inhibition of CYP3A4 by pogostone was fitted with the Lineweaver-Burk analysis, and it was found that the inhibition of CYP3A4 was non-competitive. The *K_i_* value of CYP3A4 was further obtained as 5.69 μM (Figure 2(A)). Additionally, the inhibitory effect of pogostone on the activity of CYP3A4 was affected by the incubation time, indicating the time-dependent characteristic of the inhibition of CYP3A4 by pogostone (Figure 2(B)). The KI and Kinact value of CYP3A4 were 5.86/μM and 0.056/min, respectively.

### The inhibition of CYP2C9 and 2E1 by pogostone

**Figure 3.**
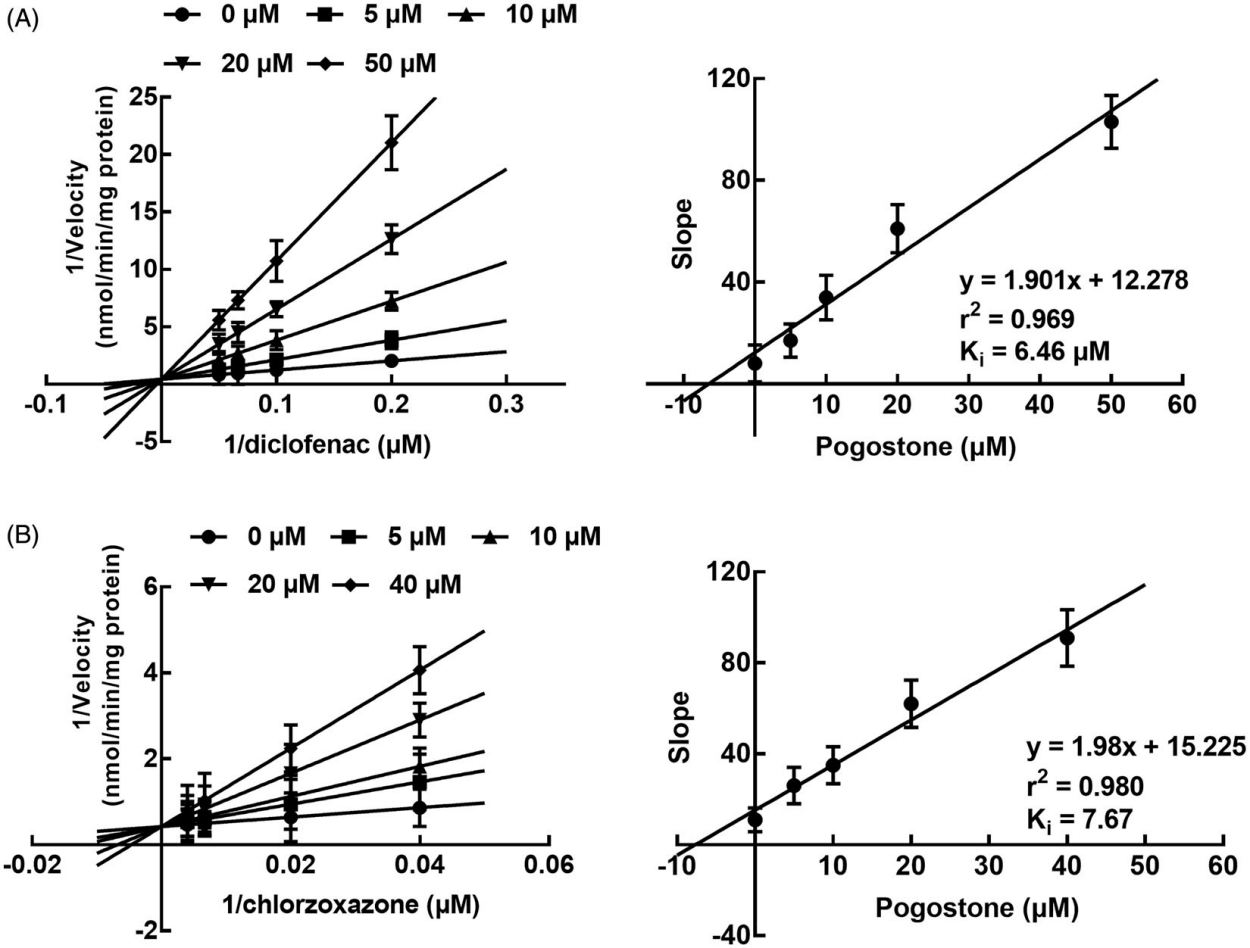
The inhibition of CYP2C9 and 2E1. (A) The Lineweaver-Burk plots of CYP2C9 in the presence of 0, 2, 5, 10, and 30 μM pogostone and 2–20 μM diclofenac. The inhibition of CYP2C9 was found to be competitive and the Ki value was obtained as 6.46 μM. (B) The Lineweaver-Burk plots of CYP2E1 in the presence of 0, 2, 5, 10, and 30 μM pogostone and 25–250 μM chlorzoxazone. The *K_i_* value was obtained as 7.67 μM.

Contrary to the inhibition of CYP3A4, the inhibition of CYP2C9 and 2E1 by pogostone was found to be competitive with the *K_i_* values of 6.46 and 7.67 μM (Figure 3). While the incubation time showed no significant effect on the inhibition of CYP2C9 and 2E1.

## Discussion

Pogostone is the major extraction of *Pogostemon cablin*, a commonly used traditional Chinese herb for vomit, diarrhoea, and headache (Swamy and Sinniah 2015; An et al. 2019). Pogostone has been demonstrated to possess favourable anti-inflammatory and antibacterial properties, which makes it used as a bacteriostat in the clinic (Li et al. 2014; Peng et al. 2014). Moreover, in recent studies, pogostone has been revealed to induce the apoptosis of human colorectal carcinoma and lung cancer cells (Cao et al. 2017; Yang et al. 2018), and protect from gastric ulcer and acute lung injury (Chen et al. 2016; Sun et al. 2016). The influence of pogostone on the activity of major isoforms of CYP450, including CYP1A2, 2A6, 3A4, 2C8, 2C9, 2C19, 2D6, and 2E1, was evaluated in this study. It was found that pogostone significantly inhibited the activity of CYP3A4, 2C9, and 2E1.

CYP3A4 is an important member of the CYP3A family, which is responsible for the biotransformation of a variety of drugs, such as paclitaxel, vincristine, and felodipine (Henningsson et al. 2005; Hendrikx et al. 2013; Topletz et al. 2013; Xiang et al. 2017). CYP3A4 has been revealed to mediate numerous drug-drug interactions. For example, amlodipine and atorvastatin are two commonly used drugs in the clinical treatment of hypertension and dyslipidemia (Curran 2010). The co-administration of these two drugs induced adverse drug-drug interaction due to the inhibition of CYP3A4 by atorvastatin (Yang et al. 2020). Here, pogostone was found to significantly inhibited the activity of CYP3A4 in a dose-dependent and non-competitive manner, which implies the potential interaction between pogostone and drugs metabolized by CYP3A4. The inhibitory effect of pogostone on the activity of CYP3A4 increased with the incubation time, suggesting the inhibition of CYP3A4 was time-dependent and incubation time should be considered in the clinical use of pogostone. Previously, time-dependent inactivators were found to possess similar functional groups or chemical structures, such as aromatic, which are included in pogostone.

Additionally, the activity of CYP2C9 and 2E1 was also dramatically inhibited by pogostone. Different from the inhibition of CYP3A4, pogostone was identified as a competitive inhibitor of CYP2C9 and 2E1. CYP2C9 and 2E1 are also responsible for the metabolism of various drugs, including losartan and acetaminophen, which are commonly used clinical drugs. Moreover, CYP2E1 was involved in the metabolism of fluoride inhalation anaesthetics, of which the high plasma concentration would induce renal toxicity and nerve injury (Schindler and Hempelmann 1998; Gentz and Malan 2001). Therefore, the inhibition of CYP2C9 and 2E1 alert the potential risk during the co-administration of pogostone and CYP2C9 and 2E1 substrates, especially fluoride inhalation anaesthetics.

Previously, Chen et al. (2013) investigated the pharmacokinetic profile of pogostone in rats with the oral administration of 5, 10, and 20 mg/kg pogostone. The *C_max_* of 5 mg/kg pogostone was 28.99 μg/mL, which is larger than the IC_50_ value of pogostone in the inhibition of CYP3A4 and close to the IC_50_ values of pogostone in the inhibition of CYP2C9, and 2E1 (Chen et al. 2013). It was suggested that it is of great possibility that the interaction occurs between pogostone and CYP3A4, 2C9, and 2E1, which needs further *in vivo* validation.

## Conclusions

This study revealed the *in vitro* inhibitory effect of pogostone on the activity of CYP3A4, 2C9, and 2E1. Pogostone was identified as a non-competitive inhibitor of CYP3A4 and a competitive inhibitor of CYP2C9 and 2E1. Meanwhile, the incubation time was proved to be a vital factor in the inhibition of CYP3A4. These results provide reference and guidance for the clinical co-administration of pogostone with other herbs or drugs.
